# Primary breast lymphoma: A mimic of inflammatory breast cancer

**DOI:** 10.1016/j.ijscr.2018.11.040

**Published:** 2018-11-22

**Authors:** Ana Melo, Sílvia Silva, Cátia Ferreira, Ricardo Pereira, Ana Esteves, Rita Abreu Marques, Herculano Moreira, Paulo Avelar

**Affiliations:** Department of General Surgery, Centro Hospitalar Trás-os-Montes e Alto Douro, Avenida da Noruega, Lordelo, 5000-508 Vila Real, Portugal

**Keywords:** Primary breast lymphoma, Breast malignancy, Combined therapy, Case report

## Abstract

**Introduction:**

Primary breast lymphoma (PBL) is a rare disease accounting for 0.04–0.5% of all breast malignancies.

**Presentation of case:**

This article reports a clinical case of a 81-year-old woman with a history of right breast erythema and edema associated to breast pain, with over two weeks duration. Physical examination demonstrated mammary asymmetry and inflammatory signs with orange peel skin. She had the right upper limb swollen and palpable axillar and supraclavicular lymph nodes. The principal suspicion was an inflammatory breast cancer. Imaging was unspecific. Core needle biopsy of an axillary lymph node and of the breast mass identified morphological and immunophenotypic features consistent with diagnosis of primary non-Hodgkin DLBCL. Patient was guided by Hematology.

**Discussion:**

The clinical and imaging ﬁndings in breast lymphoma can mimic those of breast carcinoma. Sometimes, PBL presentation is suggestive of Inflammatory breast cancer. Diagnosis depends on adequate tissue sampling for histology examination and immunophenotyping. The therapeutic management of PBL is controversial and is not clearly established, but many studies support that it is not a surgical disease and can be treated successfully with combined chemotherapy and radiotherapy.

**Conclusion:**

Breast lymphoma must be considered in the differential diagnosis of a breast lump, even in the presence of cutaneous inflammatory changes. PBL can be treated successfully with combined therapy.

## Highlights

• Primary breast lymphoma (PBL) is a rare disease accounting for 0.04–0.5% of all breast malignancies.
• The clinical and imaging ﬁndings in breast lymphoma can mimic those of breast carcinoma.
• Sometimes, PBL presentation is suggestive of Inflammatory breast cancer.
• Diagnosis depends on adequate tissue sampling for histology examination and immunophenotyping.
• The therapeutic management of PBL is controversial and is not clearly established, but many studies support that it is not a surgical disease and can be treated successfully with combined chemotherapy and radiotherapy.
• Imaging is a useful method to monitor a patient’s response to therapy.

## 1 Introduction

The incidence of non-Hodgkin lymphoma (NHL) has increased in the last decades, particularly for extra-nodal lymphomas [1]. Breast involvement by lymphoma is very rare, and it can occur as a primary breast tumor or as an extra-nodal manifestation in systemic disease [2]. Primary breast lymphoma (PBL) represents 1% of all NHL and 2% of extra-nodal lymphomas [3,4]. In addition, breast lymphoma accounts for approximately 0.04–0.5% of malignant breast tumors [5,6]. This rarity may be explained to the fact that the breast contains very little lymphoid tissue [7].

The majority of PBL are diffuse large B-cell lymphomas (DLBCL), but there are also other less frequent subtypes [8].

The clinical presentation of PBL is highly unusual which make the diagnosis a challenge. Imaging studies are less helpful in the diagnosis of this entity compared with breast carcinoma, and its diagnosis relies on surgical biopsy or fine needle aspiration cytology (FNAC) [[9], [10], [11]].

The therapeutic management of PBL is controversial and is not clearly established, but many studies support that it is not a surgical disease and can be treated successfully with combined chemotherapy and radiotherapy [2,12,13].

We report a case suspected of an inflammatory breast cancer that was diagnosed as PBL, and discuss diagnosis and management. This case report was written according to SCARE guidelines [14].

## 2 Case report

A 81 years old female presented to the Department of Surgery with a history of right breast erythema and edema associated to breast pain, with over two weeks duration and progressive worsening.

She linked the appearance of these signals with an episode of breast trauma and she denied the presence of previous breast nodules, nipple drainage, nipple retraction, fever and constitutional symptoms.

The patient was multiparous (three pregnancies and three births) and nursed all children.

She had multiple medical co-morbidities but there was no past history of breast pathology or family history of breast malignacy.

**Fig. 1:**
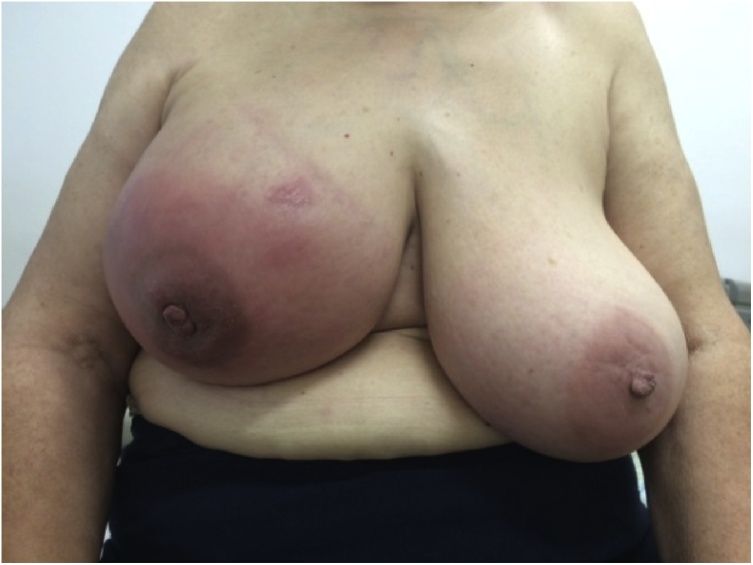
Physical examination demonstrated mammary asymmetry.

**Fig. 2:**
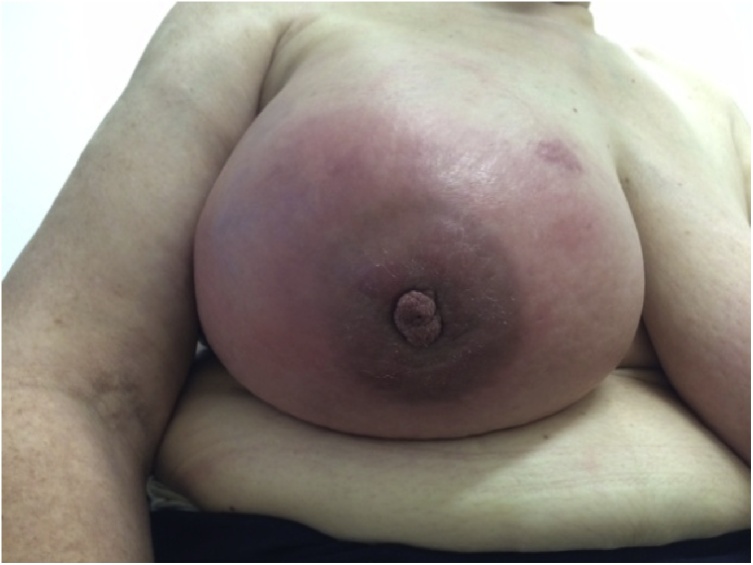
The outer quadrants and the periareolar region had inflammatory signs.

Physical examination demonstrated mammary asymmetry because right breast was bigger and tender (Fig. 1). The outer quadrants and the periareolar region had inflammatory signs with orange peel skin (Fig. 2). There was no palpable masses or nipple changes. The left breast was normal.

**Fig. 3:**
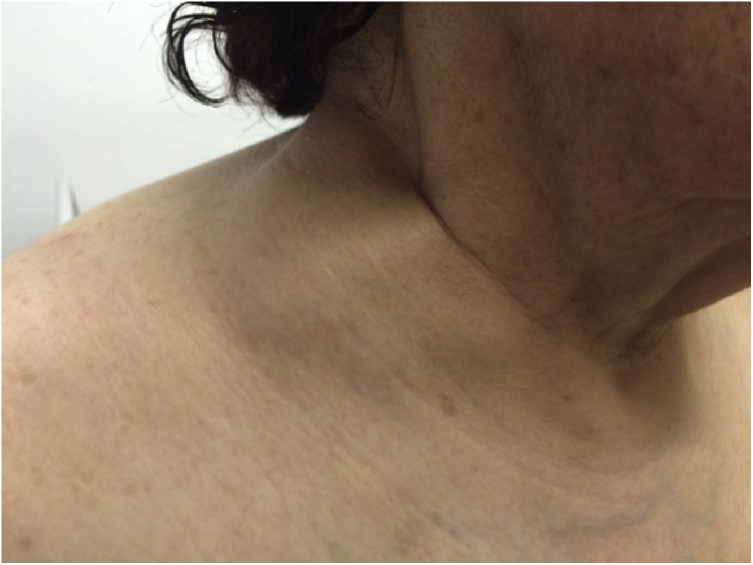
She had supraclavicular lymph nodes.

She had the right upper limb swollen and palpable axillar and supraclavicular lymph nodes (Fig. 3).

The principal suspicion was an inflammatory breast cancer.

The ultrasound of the right breast revealed skin thickening and tissue densification involving principally the external quadrants, and it was identified an irregular hypoechoic mass with 8 cm × 5 cm and multiple axillary and supraclavicular enlarged lymph nodes. The ultrasound of left breast and of left axilla was normal.

Core needle biopsy of an axillary lymph node and of the breast mass identified morphological and immunophenotypic features consistent with diagnosis of primary non-Hodgkin DLBCL.

The CT scan of the chest confirmed right breast alterations identified in ultrasound and ipsilateral axillary and supraclavicular lymph node enlargement. No other enlarged lymph nodes were observed. CT scans of the brain, abdomen and pelvis were normal. Bone marrow aspiration was negative. Serum lactate dehydrogenase level was normal.

After confirming diagnosis, the therapy plan included R-CHOP x8 (Rituximab (375 mg/m^2^, D1), cyclophosphamide (750 mg/m^2^, D1), doxorubicin (50 mg/m^2^, D1), vincristine (1.4 mg/m^2^, D1), prednisone (50 mg twice a day, D1–D5)). She also received radiotherapy (4500 cGY) to the breast and regional lymph nodes with 180 cGY daily fractions.

She is currently well at follow-up 24 months after presentation and without evidence of residual disease.

## 3 Discussion

Breast lymphoma may occur as either a primary (PBL) or a secondary (SBL) lesion [15]. The designation PBL is used when the breast is the principal or, as in most cases, the only site of lymphoma [11]. The distinction between PBL and SBL is sometimes difficult. In 1972, Wiseman and Liao first defined PBL according to four criteria: mammary tissue and lymphoma must be in close anatomic proximity, no preceding diagnosis of extra-mammary lymphoma, no evidence of disseminated disease other than ipsilateral axillary lymphadenopathy, and adequate quality of the histopathological specimen [5,16]. These criteria are still widely accepted.

The majority of patients with PBL are women; however, there are a few rare reports in males [17]. These gender differences suggest that sex hormones may be important in pathogenesis [12]. Additional support for this theory is the relatively high rate (5%–20%) of bilateral involvement at diagnosis [11,17,18].

It has a bimodal age distribution with one peak in the middle of the fourth decade and another in the seventh decade of life [19,20]. The median age of onset of non-Hodgkin breast lymphoma is 58 years [17,21]. Our patient was 81 years old woman.

Sixty percent of PBL develop in the upper outer mammary quadrant. A right sided predominance is observed with a 60:40 distribution ratio. Bilateral synchronous breast lymphoma occurs in 10% of patients and contralateral metachronous disease occurs in up to 15% of cases [17].

Patients present most frequently with a mass that mimic a carcinoma [22]. Nearly one-quarter of masses are painful, as it happened in this case. Other local signs, such as nipple retraction or discharge and skin changes, are rare [5,9]. Skin fixation and cutaneous inflammatory changes simulate inflammatory carcinoma [2]. Skin and diffuse parenchymal involvement are more common in high grade lymphomas [20,23]. Ipsilateral axillar enlarged lymph nodes have been reported in 15%–50% of cases [24,25]. In our case, the patient had all these clinical manifestations.

Likewise, systemic symptoms, such as sweating, weight loss and fever, are rare, and have been reported to occur in approximately 8–9% of the reported cases [9].

Distinct mammographic and ultrasonographic features have not been described in the literature for breast lymphoma to differentiate it from breast carcinoma or benign breast entities [9,17]. Nevertheless, imaging is a useful method to monitor a patient’s response to therapy.

Since the clinical presentation and imaging findings of breast lymphoma and carcinoma are similar, biopsy is the gold standard procedure to establish a diagnosis [26]. FNAC is a useful procedure in the diagnosis of PBL. Although its sensitivity is 90%, it has its own limitations. Most authors recommend core needle biopsy as a confirmatory method [20]. This was true in our case.

The prognosis of PBL patients depends on histologic grade and staging [5].

According to the WHO classification system for breast tumors, malignant lymphomas of the breast are subdivided into diffuse large B-cell lymphoma (DLBCL), Burkitt lymphoma, extra-nodal marginal zone (MALT) lymphoma, and follicular type. PBL are usually non-Hodgkin with a B-cell lineage [27,28]. DLBCL have worse prognosis than other histologic subtypes and have a significant higher risk of contralateral breast involvement [11].

The Wiseman-Liao definition confines PBL to Ann-Arbor IE or IIE, and patients are by definition “early stage”. The distinction between stages IE and IIE (involvement of regional lymph nodes) is important because of significant differences in 5-year overall survival (OS) for stage IE (78%–83%) compared with stage IIE (20%–57%). Bilateral PBL is still controversial with regard to stage and prognosis. The largest series of primary breast DLBCL classified cases with bilateral involvement as stage IV [11,29].

The report by the Consortium for Improving the Survival of Lymphoma (CISL) defined one extra-nodal disease (OED) category when one breast was involved and multiple extra-nodal disease (MED) when additional extra-nodal sites were present. In both categories, the presence of nodal disease was not considered relevant. The OED group had better 5-year progression-free survival (PFS) and overall survival (OS) than the MED [30].

Stratification of PBL into risk groups is usually based upon the International Prognostic Index (IPI) [28]. This model incorporates clinical features that reflect the growth and invasive potential of the tumor (tumor stage, serum DHL level, and number of extra-nodal disease sites), the patient’s response to the tumor (performance status) and the patient’s ability to tolerate intensive therapy (age and performance status) [13,20,31].

Thus, it appears that some factors consistently predict a poor outcome in primary breast DLBCL including Ann-Arbor stage > IE, poor performance status, elevated serum DHL, younger age and possibly tumor size >4–5 cm [11,17,32]. Several authors suggest that these features may be used to define higher risk PBL patients [11].

There are no current established guidelines for the treatment of PBL. Treatment options include surgery, chemotherapy and radiotherapy [2]. In general, treatment of PBL is similar to that used for other lymphomas and depends on the histological type [13]. There is no general agreement on the appropriate treatment of PBL [10]. At present, the treatment of breast lymphoma, whether primary or secondary, should be with systemic chemotherapy. The choice of chemotherapy regimen should be based upon histologic subtype, disease extent, and the individual patient [4]. Most PBL studies report favorably the administration of systemic chemotherapy even for apparently localized disease. As in nodal forms of DLBCL, an anthracycline-based regimen is the mainstay of treatment, with CHOP being the most frequent regimen used [26]. The CISL study concluded that treatment with less than four cycles of chemotherapy had a negative effect on outcome both in terms of 5-year PFS and OS [11,18]. Although not supported by robust published data, several authors favor adding rituximab to chemotherapy, in view of the better overall chance of lymphoma eradication, as well as a possible decrease in the rate of CNS relapse [11,26].

Radiation therapy alone is inadequate in controlling this disease [20]. Radiotherapy may be used as an adjuvant consolidation therapy or as a primary local therapy to increase local control. Various doses of radiation to the breast, chest wall and regional lymph nodes had been used in previous studies. The range of total doses were between 1200 and 5500 cGY and daily doses were between 180 and 300 cGY [20,29,33].

The role of surgery is minimal since tumors are highly sensitive both to chemotherapy and radiotherapy. A large meta-analysis demonstrated that radical surgery offers no benefit in this disease [8,32]. Surgery should be only for diagnostic purpose [4,29].

In conclusion, breast lymphoma must be considered in the differential diagnosis of a breast lump, even in the presence of cutaneous inflammatory changes. Diagnosis depends on adequate tissue sampling for histology examination and immunophenotyping. Nevertheless, imaging is a useful method to monitor a patient’s response to therapy. PBL is considered a non-surgical disease and can be treated successfully with combined chemotherapy and radiotherapy.

## Conflicts of interest

Nothing to state.

## Sources of funding

This research did not receive any specific grant from funding agencies in the public, commercial, or not-for-profit sectors.

## Ethical approval

Not submitted to ethical approval – Case report.

## Consent

Written informed consent was obtained from the patient for publication of this case report and accompanying images.

## Author’s contribution

Ana Melo: study design, data collection, interpretation and writing.

Ana Esteves: study concept, design and review of manuscript.

Sílvia Silva, Cátia Ferreira, Ricardo Pereira, Rita Abreu Marques: data collection and interpretation.

Herculano Moreira, Paulo Avelar: review of manuscript.

## Registration of research studies

Not applicable.

## Guarantor

Ana Esteves.

## Provenance and peer review

Not commissioned externally peer reviewed.
